# Treatment continuation of four long-acting antipsychotic medications in the Netherlands and Belgium: A retrospective database study

**DOI:** 10.1371/journal.pone.0179049

**Published:** 2017-06-14

**Authors:** Flore Decuypere, Jan Sermon, Paul Geerts, Tom R. Denee, Cedric De Vos, Bart Malfait, Mark Lamotte, Cornelis L. Mulder

**Affiliations:** 1 Commercial Services, QuintilesIMS, Zaventem, Belgium; 2 Health Economics, Market Access, Reimbursement, Janssen-Cilag NV, Beerse, Belgium; 3 Medical Affairs, Janssen-Cilag NV, Beerse, Belgium; 4 Health Economics, Market Access, Reimbursement, Janssen-Cilag BV, Tilburg, Netherlands; 5 Real-World Evidence Solutions, QuintilesIMS, Zaventem, Belgium; 6 Department of Psychiatry, Erasmus MC, Rotterdam, Netherlands; Maastricht University, NETHERLANDS

## Abstract

Achieving greater continuation of treatment is a key element to improve treatment outcomes in schizophrenia patients. However, reported treatment continuation can differ markedly depending on the study design. In a retrospective setting, treatment continuation remains overall poor among patients using antipsychotics. This study aimed to document the difference in treatment continuation between four long-acting injectable antipsychotics based on the QuintilesIMS LRx databases, national, longitudinal, panel based prescription databases of retail pharmacies, in the Netherlands and Belgium. Paliperidone palmitate once monthly, risperidone microspheres, haloperidol decanoate, and olanzapine pamoate were studied. This study demonstrated significantly higher treatment continuation of paliperidone palmitate once monthly compared to risperidone microspheres (p-value<0,01) and haloperidol decanoate (p-value<0,01) in both countries, a significantly higher treatment continuation of paliperidone palmitate once monthly compared to olanzapine pamoate in the Netherlands (p-value<0,01), and a general trend towards better treatment continuation versus olanzapine pamoate in Belgium. Analysing the subgroup of patients without previous exposure to long-acting antipsychotic treatment revealed the positive impact of previous exposure on treatment continuation with a subsequent long acting treatment. Additionally, the probability of restarting the index therapy was higher among patients treated with paliperidone palmitate once monthly compared to patients treated with risperidone microspheres and haloperidol decanoate. The data source used and the methodology defined ensured for the first time a comparison of treatment continuation in a non-interventional study design for the four long-acting injectable antipsychotics studied.

## Introduction

Treatment continuation used as an indicator of clinical effectiveness in schizophrenia informs how well a treatment performs in clinical reality [1]. Achieving greater continuation of treatment is a key element to improve treatment outcomes in schizophrenia patients. Treatment discontinuation has been determined as a reliable and strong predictor of relapse [2–5]; with each relapse having a detrimental clinical impact [6–8] as well as an important economic burden, considering the high cost of hospitalizations [9]. While a continuous treatment with antipsychotic medication has been linked to better outcomes and less risk of relapse [2–5,10], important differences are often noted between different types of drugs. The use of long-acting injectable antipsychotic medication (LAAP) has shown in many observational non-interventional studies to be associated with less relapses and lower hospitalization rates after initiation of treatment as compared to patients treated with oral antipsychotic medication (OAP) [11–14], resulting in a lower total healthcare cost [15–20].

However, reported treatment continuation can differ markedly depending on the study design [11]. In a retrospective setting, which can be considered the most “non-interventional”, as all actions took place in the past with no parties knowing of the ulterior study decision, treatment continuation remains overall poor among patients using antipsychotics. The majority of patients discontinue treatment in the first few months after initiation [14,21,22]. Retrospective study designs are more able to show the superiority of LAAP over OAP in relevant real-life situations, while less differentiation versus orals is observed in randomized controlled trials (RCT) design or prospective observational studies [11,23–25]. The main explanation proposed for such differences is the improvement of continuation to treatment, of which adherence is a major driver and which is optimized by protocol in RCTs and prospective observational studies. Alphs et al.[25], Bossie et al. [23] and Kane et al. [24] therefore recommend using a retrospective design when studying questions related to effectiveness in real clinical practice, as looking back to what already happened is by its very nature non-interventional.

Not only are differences in clinical effectiveness observed between oral and long-acting injectable drugs, there are also differences observed between the various available long-acting injectable drugs. More in particular, recent publications, using observational data, suggest that paliperidone palmitate once monthly (further referred as PP1M) can result in better treatment continuation than risperidone microspheres [26,27]. However, this observation was never tested in a large retrospective dataset.

The main objective of this study was to use real-life data from pharmacy records in order to compare the ambulatory treatment continuation of patients with schizophrenia after initiation on one of the four main LAAP used in the Netherlands and Belgium: PP1M, risperidone microspheres, haloperidol decanoate, and olanzapine pamoate. As patients with continuous treatment present a lower risk to relapse [2], this study aims at measuring the treatment continuation for post-discharge ambulatory patients only. A secondary objective was to analyse treatment following discontinuation and the rate of restart shortly after discontinuation. Finally, the dosage and combinations with other antipsychotic medications were analysed.

## Materials and methods

### Panel description

The QuintilesIMS Real-World Data Longitudinal Prescription databases (LRx), are national databases that combine transactional data from about 1400 retail pharmacies (i.e. approx. 3/4 of pharmacies) in the Netherlands, and over 1600 retail pharmacies in Belgium (i.e. approx. 1/3 of pharmacies). Only pharmaceutical products purchased in retail pharmacies are recorded and no data from the hospital pharmacies are captured. The databases contain actual prescription pickup data from pharmacy records for anonymized individual patients. The LRx databases from different countries have already been used to study questions of persistence in different therapy areas such as diabetes [28], and Hepatitis B [29]. In both countries, the four drugs analysed are dispensed to non-hospitalized patients through retail pharmacies only, even in cases when the physician prescribes the drug during a consultation held in the hospital and when the drug is administered in the hospital. The databases therefore cover appropriately the treatment purchases of ambulatory patients. As the indication for which the medication is prescribed cannot be retrieved from the LRx database and knowing the large number of off-label prescriptions for antipsychotics in general, this was verified in a QuintilesIMS dataset derived from prescribers of antipsychotics. It was confirmed that LAAP are used predominantly (> 85%) in psychosis and schizophrenia. As such, the LRx databases can provide valuable insights into the use of LAAP for their approved indications.

### Patient selection

To optimize data quality, selection criteria were defined in order to address potential missing data transmissions from pharmacies as well as with patients who only incidentally visited a pharmacy included in the panel database and therefore cannot be followed longitudinally. Only data from pharmacies which transmitted data every month were used in the Netherlands. In Belgium, a maximum of two months of missing data was allowed and within this, only one month of missing data during the same rolling 12 months. Additionally, patients were required to have at least one transaction of any drug, either the index drug or any other drug purchased, within three months following initiation in the Netherlands. In Belgium, patients with only one transaction of the index drug and with less than three units of any other drug purchased in a panel pharmacy during the 12 months preceding their first prescription of the index drug were excluded.

Patients initiating a treatment with PP1M, risperidone microspheres, haloperidol decanoate, or olanzapine pamoate were selected. To define patients as newly initiated on the drug, only those patients that had not purchased the same drug in the 12 months preceding the index treatment were included. Once patients were selected, all their transactions for 13 months after the initiation date were retrieved. Only treatments in an ambulatory setting were analysed based on the nature of LRx data, although the treatment may have been started during hospitalization prior to inclusion. Two inclusion periods were defined in both countries. The first inclusion period corresponds to the introduction of PP1M in both countries. Treatment with one of the four drugs had to be initiated between May 2011 and April 2012 in the Netherlands and between December 2011 and August 2012 in Belgium. To be able to verify time dependent effects, a second inclusion period, from October 2012 until September 2013 in the Netherlands and from September 2012 until September 2013 in Belgium was selected. No phase 4 or observational studies were ongoing in those periods that could have resulted in patients being included in these datasets.

Age was analysed based on age bands of 5 years. For each cohort, the median age band is reported.

### Treatment continuation

Patients were followed for a total of 13 months after their first prescription of the index drug. A patient was considered as continuously on treatment either until a complete stop of the treatment or until the treatment was interrupted for a period longer than the permissible gap.

The permissible gap is the maximal time period in which a patient should revisit the pharmacy to be considered as continuing the index therapy. The permissible gap was defined as:

[coverage period of 1 unit]×[number of units in the last purchase]+[grace period] (1)

The grace period reflects the additional time allowed beyond the coverage period to allow for deviations from the theoretical prescription frequency. Used grace periods and coverage periods are provided for each drug in Table 1.

**Table 1:** Treatment durations and grace periods definitions.

	PP1M	Risperidone microspheres	Haloperidol decanoate	Olanzapine pamoate
Coverage period (in days)	28	14	28	210mg: 28 300mg: 14 / 28 405mg: NL 14 / BE 28
Grace period (in days)				
*Base scenario*	28	28	28	28
*Sensitivity analysis*	60	60	60	60
*Lower-bound*	7	3	7	3 for packs of 14 days; 7 for packs of 28 days
*Upper-bound*	390	390	390	390

The time to treatment discontinuation of each patient was then calculated as:

[Time between initiation and last purchase before treatment stop]+[coverage period of 1 unit]×[number of units in the last purchase] (2)

The coverage period for each product was defined based on the summary of product characteristics (SmPC) published by the European Medicines Agency. Initial analysis of the data revealed that for all drugs, recommendations from the summary of product characteristics were followed for the majority of patients, with the exception of olanzapine pamoate in the Netherlands. In the Netherlands, analysis of the gap between two consecutive transactions of olanzapine pamoate showed that the majority of patients using 405 mg were purchasing one injection per 14 days, instead of 28 days. For that reason, the duration of 14 days was therefore used for this strength in the Netherlands only.

A grace period of 28 days was used as base case scenario. Based on the pharmacologic properties of PP1M and risperidone microspheres extrapolated by Samtani et al. [30], 28 days after coverage period of the last injection of both drugs, blood concentrations are expected to drop below therapeutically effective levels.

A sensitivity analysis on the time to treatment discontinuation was carried out by applying different grace period lengths as indicated in Table 1.

### Previous treatment and treatment following discontinuation

Therapy used before the index treatment was analysed based on transactions retrieved during the three months before initiation of the index drug. Patients were classified per type of previous treatment by order of priority: patients having purchased long-acting injectable antipsychotics were classified in the first group; among remaining patients, patients who purchased other antipsychotics were classified in the second group; finally, patients with no transaction of antipsychotics were classified in the third group.

The potential treatment started after discontinuation of the index drug was collected during the three months following discontinuation. This analysis was only carried out on patients that were receiving the index drug for less than ten months. Based on the transactions retrieved during the three months after discontinuation, patients that purchased the index drug were classified as restart. Other patients were classified in different groups of treatment using the same definitions as when analysing the treatment before initiation.

### Dosage

For patients with more than one purchase, dosage was analysed at the start and end of the observed treatment. The dosage at start was defined as the strength of the first injection purchased. If a specific initiation scheme (as defined by SmPC) is used, only the first injection obtained after the initiation period was used to determine the dose at start of treatment. The dosage at end of treatment was defined as the strength of the last injection purchased within the time of continuous medication.

Additionally, the average dosage per 28 days was calculated for each patient with more than one purchase. The average dosage per 28 days was defined as the total number of milligrams purchased during the observed time on medication, divided by the number of days on medication, multiplied by 28.

### Combination therapy

Combination with other antipsychotic medication was analysed at the first month of treatment and at the sixth month of treatment. During the first month of treatment, combined medications were analysed for all patients. During the sixth month of treatment, combined medications were only analysed for patients still on their index therapy. Combined medication included all products from the anatomical therapeutic chemical (ATC) class N5A, as defined by the Ephmra. Use of anticholinergics to treat extrapyramidal side effects was analysed based on use of trihexyphenidyl and biperiden in the Netherlands and trihexyphenidyl, biperiden and procyclidine in Belgium, being the available anticholinergics per country.

For each month, based on the purchases of patients during that month, patients were classified in two groups. Patients with no purchase of other antipsychotic medication were classified as monotherapy. Patients with purchases of other antipsychotic medication were classified as combined therapy.

For each month, the percentage of patients using anticholinergics was calculated separately.

### Statistics

Differences in time to discontinuation between patient cohorts initiated on different drugs were analysed using Kaplan-Meier analysis and a Cox proportional hazards regression, controlling for the following covariates: gender, drug strength at treatment initiation, and usage of co-medication. Hazard ratios with 95% confidence intervals were estimated. The probability of restarting treatment during the three months following discontinuation was compared between the different patient cohorts using a logistic regression model with the same covariates. Odds ratios and their 95% confidence intervals were estimated. Statistical significance was assessed with reference to an a priori α level of 0,05. All statistical tests were performed using SAS software version 9 (SAS Institute, Cary, NC, USA). Sensitivity analyses were carried out by varying the grace period (see Treatment continuation, above).

## Results

### Patient population

Cohort sizes and a description of the patient baseline demographics are represented in Table 2. The number of patients on olanzapine pamoate is substantially lower than in other cohorts in both countries and in both periods, which should be considered when drawing conclusions for this treatment group.

**Table 2:** Patient baseline demographics.

	The Netherlands				Belgium			
	PP1M	Risperidone microspheres	Haloperidol decanoate	Olanzapine pamoate	PP1M	Risperidone microspheres	Haloperidol decanoate	Olanzapine pamoate
**First period**	**May11-Apr12**				**Dec11-Aug12**			
N	624	540	333	73	481	302	222	43
Gender								
*Men*	383 (61%)	334 (62%)	202 (61%)	55 (75%)	291 (60%)	155 (51%)	91 (41%)	23 (53%)
*Women*	239 (38%)	203 (38%)	116 (35%)	17 (23%)	182 (38%)	135 (45%)	119 (54%)	20 (47%)
*Unknown gender*	2 (0%)	3 (1%)	15 (5%)	1 (1%)	8 (2%)	12 (4%)	12 (5%)	0 (0%)
Age								
*Median age band*	38–42	38–42	38–42	38–42	38–42	48–52	68+	48–52
Previous medication in ambulatory setting								
*Other long-acting injectable antipsychotics*	199 (32%)	13 (2%)	9 (3%)	2 (3%)	187 (39%)	12 (4%)	6 (3%)	1 (2%)
*Other oral antipsychotics*	117 (19%)	133 (25%)	95 (29%)	24 (33%)	114 (24%)	92 (30%)	74 (33%)	16 (37%)
*No antipsychotic medication*	308 (49%)	394 (73%)	229 (69%)	47 (64%)	180 (37%)	198 (66%)	142 (64%)	26 (60%)
**Second period**	**Oct12-Sep13**				**Sep12-Sep13**			
N	524	411	373	92	813	568	401	136
Gender								
*Men*	349 (67%)	270 (66%)	205 (55%)	70 (76%)	433 (53%)	241 (42%)	165 (41%)	76 (56%)
*Women*	172 (33%)	134 (33%)	153 (41%)	20 (22%)	332 (41%)	277 (49%)	206 (51%)	44 (32%)
*Unknown gender*	3 (1%)	7 (2%)	15 (4%)	2 (2%)	48 (6%)	50 (9%)	30 (7%)	16 (12%)
Age								
*Median age band*	38–42	28–32	28–32	33–37	38–42	48–52	63–67	43–47
Previous medication in ambulatory setting								
*Other long-acting injectable antipsychotics*	76 (15%)	7 (2%)	14 (4%)	2 (2%)	128 (16%)	25 (4%)	22 (5%)	17 (13%)
*Other oral antipsychotics*	106 (20%)	109 (27%)	112 (30%)	28 (30%)	235 (29%)	149 (26%)	130 (32%)	35 (26%)
*No antipsychotic medication*	342 (65%)	295 (72%)	247 (66%)	62 (67%)	450 (55%)	394 (69%)	249 (62%)	84 (62%)

Patients originated from 586 distinct pharmacies in the Netherlands and 1105 distinct pharmacies in Belgium.

In the Netherlands, the age distribution did not differ greatly between the four drugs studied. However, in Belgium, median age was in both periods remarkably higher for patients initiated on haloperidol decanoate. Verification in the independent prescription database showed that long-acting antipsychotics are mostly (>85%) used in psychosis and schizophrenia in Belgium, so it is unlikely that this is due to usage in dementia patients.

Previous medication can also be found in Table 2. A limited percentage of patients were previously treated with other long-acting injectable antipsychotics. This percentage is higher among cohorts initiated on PP1M. Previous treatment in those cases were mostly involving risperidone microspheres.

The percentage of patients with no previous antipsychotic medication in ambulatory setting varied between 49% and 73% among the different cohorts in the Netherlands, and between 37% and 69% in Belgium.

### Treatment continuation

**Fig 1:**
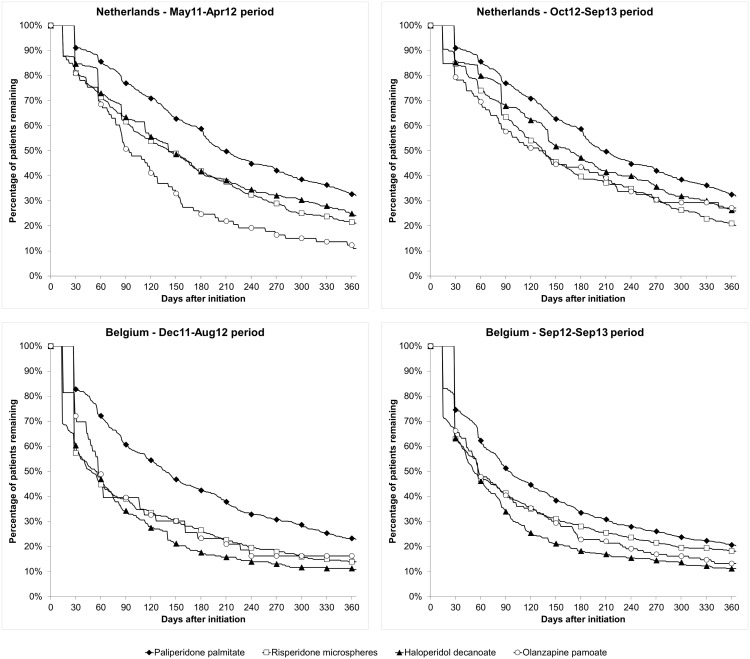
Time to treatment discontinuation curves—Base case analysis.

The time to treatment discontinuation curves are presented in Fig 1. In both periods and both countries, more patients were able to continue their index drug when initiated on PP1M than with risperidone microspheres, olanzapine pamoate or haloperidol decanoate. However, for all treatment groups, observed continuation rates were below 50% after 12 months of follow up in an ambulatory setting.

Detailed results of the time to treatment discontinuation and of the Cox regression analysis can be found in Table 3.

**Table 3:** Treatment continuation.

	The Netherlands				Belgium			
	PP1M	Risperidone microspheres	Haloperidol decanoate	Olanzapine pamoate	PP1M	Risperidone microspheres	Haloperidol decanoate	Olanzapine pamoate
**First period**	**May11-Apr12**				**Dec11-Aug12**			
N	624	540	333	73	481	302	222	43
Percentage of patients remaining on treatment								
*After one injection*	91%	88%	85%	88%	83%	69%	62%	81%
*After 6 months*	59%	42%	42%	25%	43%	26%	18%	23%
*After 12 months*	31%	21%	24%	11%	23%	14%	11%	14%
Cox regression analysis (base case)								
*Hazard ratio*	Reference	1,50	1,32	2,12	reference	1,60	1,63	1,27
*p-value*	reference	<0,01	<0,01	<0,01	reference	<0,01	<0,01	0,33
Cox regression analysis (subgroup without patients previously treated with LAAP)								
N	425	527	324	71	294	290	216	42
*Hazard ratio*	Reference	1,29	1,03	2,12	reference	1,35	1,31	1,00
*p-value*	Reference	<0,01	0,75	0,01	reference	<0,01	0,01	1,00
**Second period**	**Oct12-Sep13**				**Sep12-Sep13**			
N	524	411	373	92	813	568	401	136
Percentage of patients remaining on treatment								
*After one injection*	88%	91%	85%	85%	75%	72%	65%	83%
*After 6 months*	55%	40%	47%	43%	33%	28%	18%	23%
*After 12 months*	33%	20%	26%	27%	20%	18%	11%	13%
Cox regression analysis (base case)								
*Hazard ratio*	Reference	1,51	1,19	1,49	reference	1,37	1,45	1,26
*p-value*	Reference	<0,01	<0,01	<0,01	reference	<0,01	<0,01	0,14
Cox regression analysis (subgroup without patients previously treated with LAAP)								
N	448	404	359	90	685	543	379	119
*Hazard ratio*	Reference	1,29	1,02	1,09	reference	1,28	1,36	1,17
*p-value*	Reference	<0,01	0,83	0,70	reference	<0,01	<0,01	0,33

In the Netherlands, in both periods, the Cox regression analysis showed a statistically significant longer time to treatment discontinuation for the PP1M cohort compared to the risperidone microspheres, the haloperidol decanoate, as well as the olanzapine pamoate cohorts.

In Belgium, in both periods, the Cox regression analysis showed a statistically significant longer time on treatment for the PP1M cohort compared to the risperidone microspheres, as well as the haloperidol decanoate cohorts. The olanzapine pamoate cohorts showed a numerical but not significant higher risk for treatment discontinuation compared with the PP1M cohorts.

In both countries, as shown in Table 2, a part of the patients starting a treatment with PP1M were previously treated with other LAAP, while it was the case for only a very limited number of patients starting treatment with one of the other drugs. In order to understand the impact of this variable on the treatment continuation, a second Cox regression analysis was carried out on the subgroup of patients who were not previously treated with other LAAP. The time to treatment discontinuation curves for those cohorts are presented in supporting information (S1 Fig). Due to the low number of patients, it was not possible to carry out the same analysis on the subgroup of patients who were previously treated with other LAAP.

As presented in Table 3, the subgroup of patients not previously treated with other LAAP still showed numerical longer time to treatment discontinuation of PP1M compared to the other three drugs, but the difference was less pronounced, either by a lower hazard ratio estimation or a higher p-value. The difference in time to treatment discontinuation was significant only versus risperidone microspheres in both countries and both periods, versus haloperidol decanoate in Belgium in both periods, and versus olanzapine pamoate in the Netherlands in the May11-Apr12 period.

### Treatment following discontinuation

Table 4 summarizes the treatments received by patients during the first 3 months after discontinuation of their index drug and the results of the logistic regression model.

**Table 4:** Treatment following discontinuation.

	The Netherlands				Belgium			
	PP1M	Risperidone microspheres	Haloperidol decanoate	Olanzapine pamoate	PP1M	Risperidone microspheres	Haloperidol decanoate	Olanzapine pamoate
**First period**	**May11-Apr12**				**Dec11-Aug12**			
N	385	405	234	62	343	254	196	36
Treatment following discontinuation								
*Restart initial drug*	161 (42%)	141 (35%)	62 (26%)	27 (44%)	142 (41%)	68 (27%)	33 (17%)	9 (25%)
*Other long-acting injectable antipsychotics*	47 (12%)	39 (10%)	15 (6%)	3 (5%)	24 (7%)	5 (2%)	6 (3%)	0 (0%)
*Other oral antipsychotics*	65 (17%)	87 (21%)	63 (27%)	12 (19%)	44 (13%)	47 (19%)	44 (22%)	6 (17%)
*No antipsychotic medication*	112 (29%)	138 (34%)	94 (40%)	20 (33%)	133 (39%)	134 (53%)	113 (58%)	21 (58%)
Logistic regression model								
*Odds ratio*	reference	0,70	0,43	0,82	reference	0,71	0,46	0,45
*Confidence interval*	reference	0,54–0,90	0,31–0,58	0,52–1,30	reference	0,49–1,02	0,29–0,74	0,13–1,62
*p-value*	reference	<0,01	<0,01	0,39	reference	0,07	<0,01	0,22
**Second period**	**Oct12-Sep13**				**Sep12-Sep13**			
N	320	303	254	65	622	457	350	114
Treatment following discontinuation								
*Restart initial drug*	128 (40%)	101 (33%)	72 (28%)	27 (42%)	228 (37%)	109 (24%)	75 (21%)	39 (34%)
*Other long-acting injectable antipsychotics*	15 (5%)	29 (10%)	8 (3%)	6 (9%)	27 (4%)	26 (6%)	19 (5%)	7 (6%)
*Other oral antipsychotics*	49 (15%)	62 (20%)	73 (29%)	17 (26%)	84 (14%)	60 (13%)	52 (15%)	20 (18%)
*No antipsychotic medication*	128 (40%)	111 (37%)	101 (40%)	15 (23%)	283 (45%)	262 (57%)	204 (58%)	48 (42%)
Logistic regression model								
*Odds ratio*	reference	0,80	0,58	0,89	reference	0,50	0,47	0,68
*Confidence interval*	reference	0,61–1,06	0,43–0,79	0,56–1,42	reference	0,38–0,68	0,33–0,65	0,39–1,32
*p-value*	reference	<0,01	0,12	0,63	reference	<0,01	<0,01	0,25

In the Netherlands, more patients restarted their index drug among patients treated with PP1M compared to risperidone microspheres and haloperidol decanoate, while numbers were similar for olanzapine pamoate. Significantly lower probabilities to restart index drug compared to PP1M were found with risperidone microspheres cohort and the haloperidol decanoate cohort in the May11-Apr12 period, whereas in the Oct12-Sep13 period, the risperidone microspheres cohort showed a significantly lower probability to restart index drug compared to PP1M.

In Belgium, within the first 3 months after discontinuation, more patients restarted their index drug among patients treated with PP1M compared to risperidone microspheres, haloperidol decanoate, and olanzapine pamoate. In the Dec11-Aug12 period, compared to PP1M, the haloperidol decanoate cohort showed a significantly lower probability to restart index drug. In the Sep12-Sep13 period, the risperidone microspheres cohort and the haloperidol decanoate cohort showed a significantly lower probability to restart index drug.

In both countries and in both periods, between 23% and 58% of patients did not purchase any antipsychotic medication in the pharmacy during the 3 months following discontinuation.

### Sensitivity analysis

**Fig 2:**
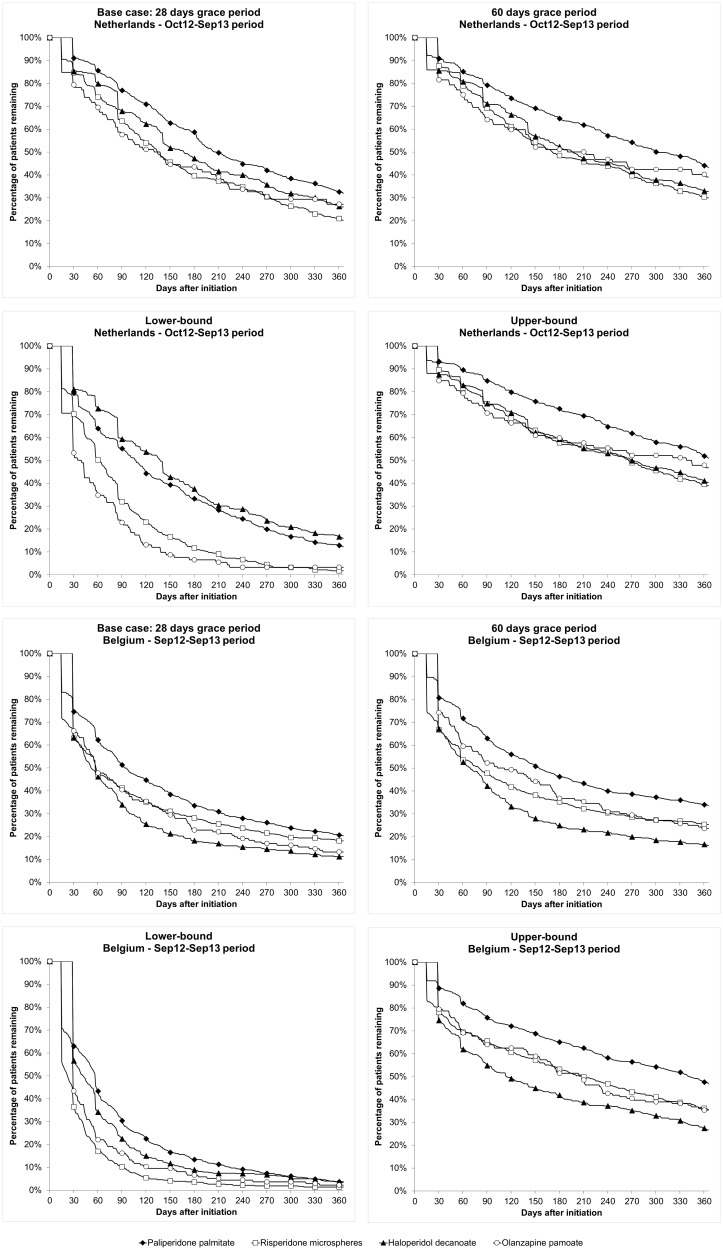
Time to treatment discontinuation sensitivity analysis.

The definition of the grace period has a significant impact on the discontinuation rate. Therefore, a sensitivity analysis has been conducted to understand the impact of the length of the permissible gap. The sensitivity analysis on the time to treatment discontinuation for patients treated with PP1M, risperidone microspheres, haloperidol decanoate and olanzapine pamoate is presented in Fig 2. Only the results of the second inclusion period are presented here, as these are similar to those of the first inclusion period.

In all grace period scenarios, the percentage of patients continuing treatment with PP1M is numerically higher than the percentage of patients continuing treatment with the three other drugs, except for haloperidol decanoate in the Netherlands using the lower-bound scenario. Detailed results can be found in supporting information (S1 Table).

### Dosage

The median dose per 28 day period was 90–100 mg for PP1M, 72–78 mg for risperidone microspheres, 97–101 mg for haloperidol decanoate and 406–572 mg for olanzapine pamoate. The modal start dose equalled modal end dose in most cases. A reduction between mode start and end dose was found for PP1M in the May11-Apr12 cohort in the Netherlands and for olanzapine pamoate in the Sep12-Sep13 cohort in Belgium. Few patients, less than 15% in all cases, increased the initial dose. Mode and median dosing are presented in supporting information (S2 Table).

### Combination therapy

Antipsychotic monotherapy was the norm for all products, as more than 50% of patients used the product in monotherapy.

Use of medication to treat extrapyramidal side effects after 6 months of therapy with PP1M, risperidone microspheres, haloperidol decanoate and olanzapine pamoate was respectively 6%, 15%, 26% and 7% in the Netherlands during May11-Apr12 period and 8%, 6%, 22% and 5% during Oct12-Sep13 period. For Belgium these data were 12%, 26%, 52% and 15% during Dec11-Aug12 period and 7%, 7%, 20% and 8% during Oct12-Sep13 period.

## Discussion

This study aimed to document the difference in treatment continuation between four LAAP, based on data provided by national, longitudinal, panel based prescription databases of retail pharmacies in the Netherlands and in Belgium. This retrospective study demonstrated a significantly higher treatment continuation of PP1M compared to risperidone microspheres and haloperidol decanoate in both countries, a significantly higher treatment continuation of PP1M compared to olanzapine pamoate in the Netherlands, and a general trend towards better treatment continuation versus olanzapine pamoate in Belgium. Analysing the subgroup of patients without previous exposure to long-acting antipsychotic treatment revealed a positive impact of previous exposure on treatment continuation with a next long acting treatment. This subgroup analysis demonstrated a significantly higher treatment continuation of PP1M compared to risperidone microspheres and a general trend towards better treatment continuation versus haloperidol decanoate and olanzapine pamoate, even without previous exposure to long-acting antipsychotic treatment. Additionally, the probability of restarting the index therapy was higher among patients treated with PP1M compared to patients treated with risperidone microspheres and haloperidol decanoate. The low number of patients in the olanzapine pamoate cohort might have affected the statistical analysis so that only a numerical improvement in treatment continuation and no difference in index therapy restart rate were found between PP1M versus olanzapine pamoate.

This study demonstrates differences in post discharge treatment continuation, specifically in the first year of a newly started long-acting medication. While it had already been demonstrated that long-acting antipsychotics perform better than orals in delaying relapse in real-world situations [11,12], this study shows that there also exist differences among long-acting antipsychotics in such real-world circumstances. Although we found that PP1M performed better than other long acting medications, the clinical significance of this finding needs further investigation, especially given the usual long periods of psychotic disorder over a patient lifetime.

On the other hand, in view of the expected channel bias that directed the most severe patients to the newest medication and the limited available covariates that enable to adjust for such cohort effects, it is somewhat unexpected that significant differences could be identified between long-acting treatments with this type of analysis. As a result, although the observed differences in treatment duration are modest, they do indicate that incremental treatment extensions between long-acting drugs are possible. This is clinically relevant as continuation of treatment is a major influence factor on the risk of relapse and discontinuation of medication increases the relapse risk by a factor of 6 [2]. It remains then an important clinical treatment aim to reduce the risk of relapse as much as possible, for several reasons. Both the number [4,6,31–35] and total duration of subsequent relapses [36] have been linked with worsening of clinical indicators such as treatment resistance and is, for the patient, not conductive to continuous functioning nor autonomy [8,9,36–39]. One could therefore argue that any relapse delay is worth the effort from a clinical perspective, and also because it is known that relapses drive costs through hospitalization cost which is by far the major cost element of schizophrenia [9,40]. It would be beneficial to perform additional research over longer time periods to further investigate the clinical benefit of treatment duration extension. In such research, continuous longitudinal follow-up during periods when the patient is not exposed to medication should also be included as recent research has shown that a longer half-life of long acting treatments might result in longer relapse prevention after treatment discontinuation [41]. A growing amount of data of clinical [42–44], local observational [45–47], product and patient related factors feed the rationale that PP1M can result in better treatment continuation compared to risperidone microspheres. However, this hypothesis had not been investigated in the context of a large retrospective real-world database. The intuitively most obvious reason for helping patients to maintain treatment long-term is a monthly versus biweekly administration. In a recent study determining the utility of administration interval in schizophrenia as judged by the general population, Osborne et al. [48] found a statistically significant advantage (p<0,001) in utility value of monthly (mean utility 0,65) versus biweekly (mean utility 0,61) administration interval. In addition, differences in convenience and ease of administration might help as well. Different formulation technology for PP1M resulted in smaller needle size and increased ease of preparation and storage.

Confirming the results of other studies [22,49], the overall treatment continuation after 1 year remains low. A large number of patients already discontinue in the first few months of treatment, especially in Belgium.

A first important consideration to explain these findings is that treatment continuation rates differ depending on the study setting. Typically, retrospective studies demonstrate lower treatment continuation than prospective studies, because, although even if non-interventional, there is still the sense of the controlled trial environment, including the contact with the study center [14,21,22].

Additionally, methodological elements influence the results. All prescription interruptions longer than the permissible gap period were considered as treatment discontinuations, irrespective of the reason for the discontinuation. As the reasons for discontinuation are not available in the database used, these cannot be investigated. It is also possible that in some cases the treatment is not ‘de facto’ discontinued, but the patient is, for example, hospitalized. This is however potentially also a measure of treatment failure and therefore another marker of effectiveness. Any disappearance or interruption beyond the permissible gap period is considered as a discontinuation in this analysis while the reason is not known. While the above limitations are a consequence of the database used, methodological parameters like the length of the ‘grace period’ have important consequences as well. The impact of the “grace period”, i.e. how long a period is accepted after the day the patient should have picked up the next administration was evaluated in a sensitivity analysis. While careful consideration was given in the selection of the permissible gap period of the primary analysis (see Materials and methods), more pragmatic and longer periods might be considered acceptable in clinical practice. Therefore the sensitivity analyses reflected grace periods of 60 days to 13 months like those applied in other studies [50,51]. By applying the most stringent parameters in the primary analysis, this study focused on highly persistent patients, as short time treatment discontinuations are suspected to cause detrimental negative outcomes [52]. One-year continuation rate following these stringent criteria are considered low. However, continuation rates naturally increased with increasing grace periods. When the grace period was put more liberal, what could be considered as more in line with clinical practice, one-year continuation data approached results of prospective observational trials. But more importantly, the ranking of the diverse products in terms of continuation remains consistent and overall results of the primary analysis were confirmed.

Furthermore, it should also be considered that the patient population could represent a study cohort that is inherently advanced in the severity of their disease. More than 50% of the patients were not ambulatory treated before a LAAP therapy was started. Following therapy discontinuation, again 23% to 58% of patients were not visiting the ambulatory pharmacy any more, although they were visiting a pharmacy while the treatment was ongoing. Additionally, an important group of the PP1M patients were using long acting medication before they started the PP1M treatment, indicating the selection bias that occurs when a new product becomes available: the more advanced patients are being prioritized to the newest available medication. On the other hand, it must be considered that relatively young patients were also included in most groups.

Mode start and end dose were well within registered label except for olanzapine pamoate. When calculating the amount of drug consumed in a 28 day period for comparison reasons, some discrepancies occur. Risperidone microspheres at 72–78 mg median dose over 28 days in our study is representative of the recalculated DDD of 75,6 mg per 28 days. Haloperidol decanoate is slightly higher at 97–101 mg versus 92,4mg, and PP1M somewhat higher at 93–100 mg versus 75 mg. The greatest discrepancy is olanzapine pamoate at 406–572 mg versus 280 mg DDD. We found no evidence of so-called “dose creep”, as dose increases during the study period were limited to less than 15% of all patients for all products, varying from 0 to 14% without any visible correlation to country or product.

The remarkably consistent high and generally increasing share of antipsychotic monotherapy probably reflects the ambulatory nature of the sample. Anticholinergic use reflected the known pharmacological profiles of the drugs, most notably a higher use of medication to treat extrapyramidal symptoms with haloperidol decanoate.

Other direct comparisons between long-acting antipsychotics are few. A recent comparison of haloperidol decanoate and PP1M [53] found no difference in efficacy. However, this study has been criticized on the account of its high attrition rate, unusual and potentially subjective measure of “lack of efficacy”, non-equivalent dosing, unclear reporting as well as lack of statistical power [54–56].

Based on the outcomes of the present study, a potential next step might be to develop a health-economical evaluation study which assesses the health-economical outcomes of the included long-acting antipsychotics. Recent publications have shown PP1M to be either economically dominant (better effectiveness for lower cost) or cost effective [17,19,57–61] versus risperidone microspheres, olanzapine pamoate, haloperidol decanoate, zuclopentixol decanoate or a selection of oral antipsychotics.

Limitations linked to the databases used in this study should be taken into account when interpreting the results. Firstly, the LRx databases do not provide information on how the patients use the drugs or on the doctor’s decision to treat. Analysed treatment episodes of patients are only based on the transactions captured at the pharmacy which are taken as a proxy for actual use of the product. Continuation of successive transactions is taken as a proxy for treatment continuation. It may be assumed that if a patient continues to pick up the product at the pharmacy, he or she is using it, and that any error size is common to all products. When a patient discontinues treatment, it is also not known whether the patient continues consulting his doctor, whether the patient still received a prescription or whether the doctor decided not to prescribe. Assumptions on the start date of treatment and on the treatment duration of each pack purchased therefore needed to be made in order to carry out this study. However, a limited impact is expected from those assumptions as these are considered to be equal for all drugs studied. In addition, causal relationships cannot be investigated in this study. In this analysis, the four groups were not identical at the start of the observation period due to the observational retrospective design of the study. This is particularly important with respect to the type of previous medications used. As such baseline differences (e.g. the potential channel bias that new products are used to treat the most severe patients first) might occur, we analysed the subgroup of patients that had no previous exposure to long-acting treatment before starting the index treatment.

Secondly, the most important limitation is linked to the loyalty of patients to their pharmacy. Patients can indeed visit several pharmacies. This is the case when a patient moves, but also when a patient occasionally visits another pharmacy than his or her usual pharmacy, or when a patient regularly visits several pharmacies. In the Netherlands, a unique patient identifier is not in place, which means that a patient who visited two different pharmacies in the panel during the follow-up period is limited to the first exposure period only. In Belgium, when a patient changes pharmacy within the panel, there will be no breach in the longitudinal prescription tracking, as a result of a unique patient identifier in all pharmacies of the LRx panel. However, if a patient visits a pharmacy in the LRx panel and another out of the LRx panel, only the first sequence of transactions will be considered in the longitudinal tracking. The impact of incidental off-panel visits is limited by the grace period and the sensitivity analyses. Eligibility rules have been defined in order to limit the impact of this phenomenon on the results. However, not all cases of patients changing pharmacy can be detected. As a result, treatment continuation might have been underestimated. This underestimation cannot be quantified, but is believed to be limited as a result of a high loyalty among chronic patients to their pharmacy [62]. Moreover, loyalty to a pharmacy is not dependent on the antipsychotic used; therefore impact should be similar for all drugs in this study. A reasonable assumption might be that, due to the high stigma of using antipsychotics and of psychosis itself, patients prefer the pharmacy that they feel most comfortable with.

The final limitation is related to the continuity in the selection of pharmacies transmitting data to QuintilesIMS. It is possible that a pharmacy does not transmit data or partially transmits data for a specific month. The transactions of patients visiting those pharmacies during those months are then missing. Eligibility rules on pharmacies have therefore been defined in order to monitor data transmissions and select pharmacies for which a sufficient continuity in the transmissions is observed. Furthermore, long-acting antipsychotics were verified in a prescription-oriented database by QuintilesIMS to be mostly (>85%) used in psychosis and schizophrenia.

While those limitations can impact the measurement of the treatment continuation, it is important to note that none of the limitations of the LRx databases are believed to impact the comparison of treatment continuation between the four long-acting antipsychotic drugs studied.

Differences in terms of the structure of the database, the number of pharmacies that were providing data as well as the follow-up care for patients with schizophrenia in both countries make it impossible to draw robust conclusions when comparing the datasets in both countries.

In conclusion, this study demonstrates that patients stay significantly longer on treatment when initiated on PP1M compared to risperidone microspheres and haloperidol decanoate. It also indicated a higher treatment continuation of PP1M over olanzapine pamoate in the Netherlands. Additionally, it shows the positive impact of previous exposure to long-acting treatments on treatment continuation with a next long acting treatment. Despite some limitations, the data source used and the methodology defined ensure a reliable comparison between the four products in a real-world setting. As such, this study provides for the first time a comparison of the treatment continuation in a large non-interventional study design for the four long-acting injectable antipsychotics: PP1M, risperidone microspheres, haloperidol decanoate, and olanzapine pamoate.

## Supporting information

S1 Fig Time to treatment discontinuation curves—Excluding patients previously treated with long-acting antipsychotics. (EPS)

S1 Table Time to treatment discontinuation sensitivity analysis. (DOCX)

S2 Table Dosage at start and end of treatment and median dosage per 28 day. (DOCX)
