# Supplementary material for: Treatment continuation of four long-acting antipsychotic medications in the Netherlands and Belgium: A retrospective database study
Source: PLoS One. 2017 Jun 14;12(6):e0179049. doi: 10.1371/journal.pone.0179049 (PMC5470699; doi:10.1371/journal.pone.0179049)
Supplement: S1 Table — (DOCX) [file pone.0179049.s002.docx]

S1 Table. Time to treatment discontinuation sensitivity analysis

|  |  |  | | | **The Netherlands** | | | | | | |  | **Belgium** | | | |
| --- | --- | --- | --- | --- | --- | --- | --- | --- | --- | --- | --- | --- | --- | --- | --- | --- |
|  |  |  | | | Paliperidone palmitate | Risperidone microspheres | | | Haloperidol decanoate | Olanzapine pamoate | |  | Paliperidone palmitate | Risperidone microspheres | Haloperidol decanoate | Olanzapine pamoate |
| **Second period** | | | | | **Oct12-Sep13** | | | | | | |  | **Sep12-Sep13** | | | |
|  |  | | N | 524 | | | 411 | 373 | | | 92 |  | 813 | 568 | 401 | 136 |
|  | Percentage of patients remaining on treatment | | | | | | | | | | |  |  |  |  |  |
|  |  | *After one injection* | | | 91% | 92% | | | 86% | 86% | |  | 81% | 75% | 68% | 90% |
|  |  | *After 6 months* | | | 65% | 48% | | | 52% | 51% | |  | 46% | 35% | 25% | 37% |
|  |  | *After 12 months* | | | 43% | 30% | | | 33% | 39% | |  | 34% | 25% | 16% | 24% |
|  | Percentage of patients remaining on treatment - Lower-bound | | | | | | | | | | |  |  |  |  |  |
|  |  | *After one injection* | | | 79% | 81% | | | 81% | 71% | |  | 63% | 56% | 59% | 71% |
|  |  | *After 6 months* | | | 33% | 12% | | | 38% | 7% | |  | 13% | 4% | 9% | 7% |
|  |  | *After 12 months* | | | 12% | 2% | | | 16% | 3% | |  | 3% | 1% | 4% | 2% |
|  | Percentage of patients remaining on treatment - Upper-bound | | | | | | | | | | |  |  |  |  |  |
|  |  | *After one injection* | | | 93% | 94% | | | 87% | 88% | |  | 89% | 83% | 75% | 92% |
|  |  | *After 6 months* | | | 73% | 58% | | | 59% | 60% | |  | 65% | 53% | 42% | 51% |
|  |  | *After 12 months* | | | 51% | 39% | | | 40% | 47% | |  | 47% | 35% | 27% | 35% |
