# Supplementary material for: Treatment continuation of four long-acting antipsychotic medications in the Netherlands and Belgium: A retrospective database study
Source: PLoS One. 2017 Jun 14;12(6):e0179049. doi: 10.1371/journal.pone.0179049 (PMC5470699; doi:10.1371/journal.pone.0179049)
Supplement: S2 Table — (DOCX) [file pone.0179049.s003.docx]

S2 Table. Dosage at start and end of treatment and median dosage per 28 days

|  |  |  | **The Netherlands** | | | |  | **Belgium** | | | |
| --- | --- | --- | --- | --- | --- | --- | --- | --- | --- | --- | --- |
|  |  |  | Paliperidone palmitate | Risperidone microspheres | Haloperidol decanoate | Olanzapine pamoate |  | Paliperidone palmitate | Risperidone microspheres | Haloperidol decanoate | Olanzapine pamoate |
| **First period** | | | **May11-Apr12** | | | |  | **Dec11-Aug12** | | | |
|  |  | N | 554 | 415 | 227 | 61 |  | 390 | 164 | 138 | 30 |
|  | Dosage | |  |  |  |  |  |  |  |  |  |
|  |  | *Mode start dosage* | 100mg (37%) | 25mg (38%) | 50mg (55%) | 300mg (56%) |  | 100mg (39%) | 50mg (36%) | 50mg (57%) | 405mg (60%) |
|  |  | *Mode end dosage* | 75mg (35%) | 25mg (37%) | 50mg (57%) | 300mg (51%) |  | 100mg (35%) | 50mg (37%) | 50mg (51%) | 405mg (50%) |
|  |  | *Percentage of patients increasing dosage* | 14% | 12% | 0% | 10% |  | 12% | 12% | 10% | 7% |
|  |  | *Median dosage per 28 days* | 90mg | 77mg | 97mg | 572mg |  | 93mg | 78mg | 100mg | 421mg |
| **Second period** | | | **Oct12-Sep13** | | | |  | **Sep12-Sep13** | | | |
|  |  | N | 437 | 323 | 269 | 70 |  | 588 | 392 | 228 | 93 |
|  | Dosage | |  |  |  |  |  |  |  |  |  |
|  |  | *Mode start dosage* | 75mg (34%) | 25mg (38%) | 50mg (52%) | 300mg (46%) |  | 100mg (37%) | 25mg (42%) | 50mg (50%) | 405mg (45%) |
|  |  | *Mode end dosage* | 75mg (33%) | 25mg (38%) | 50mg (51%) | 300mg (37%) |  | 100mg (37%) | 25mg (38%) | 50mg (56%) | 300mg (41%) |
|  |  | *Percentage of patients increasing dosage* | 10% | 11% | 7% | 7% |  | 11% | 8% | 6% | 12% |
|  |  | *Median dosage per 28 days* | 96mg | 76mg | 101mg | 561mg |  | 100mg | 72mg | 100mg | 406mg |
